# Dietary Sources of Glycine Betaine and Proline Betaine in Plant Foods and Their Potential Biological Relevance in Human Nutrition

**DOI:** 10.3390/foods15040759

**Published:** 2026-02-19

**Authors:** Bruna Laratta, Rosanna Squitti, Domenico Cautela

**Affiliations:** 1 National Research Council (CNR), Institute of Biosciences and Bio-Resources (IBBR), Via P. Castellino 111, 80131 Naples, Italy; bruna.laratta@cnr.it; 2 Department of Theoretical and Applied Sciences (DiSTA), E-Campus University, 22060 Novedrate, Italy; rosanna.squitti@uniecampus.it

**Keywords:** glycine betaine (GlyBet), proline betaine (stachydrine; ProBet), LC–MS quantification, nutritional biomarkers

## Abstract

Betaines are natural nitrogen-containing compounds widely distributed in plant-derived foods and animal tissues, where they function primarily as osmolytes, chaperons, and methyl donors. As such, they have attracted increasing interest as dietary components and metabolic biomarkers in human nutrition. This study provides a comparative characterization of glycine betaine (GlyBet) and proline betaine (ProBet) by combining targeted LC–MS quantification in a representative selection of plant-based foods with complementary in silico analyses and integration of dietary intake estimates derived from published nutritional and metabolomic studies, together with human metabolomic data. A validated HPLC–ESI–MS method was applied to quantify GlyBet and ProBet across cereals, pseudocereals, vegetables, and fruits. GlyBet was found to be predominantly abundant in leafy vegetables and in several cereal and pseudocereal flours, whereas ProBet was highly enriched in citrus fruits, particularly bergamot, chinotto, and bitter orange. In silico ADMET predictions were used to provide a qualitative and comparative description of the pharmacokinetic and safety-related properties of the two betaines, indicating broadly similar hydrophilic profiles with modest differences in solubility, clearance, and predicted skin sensitization. Similarity-based target prediction analyses, used in an exploratory framework, suggest distinct contextual tendencies for the two betaines. GlyBet is primarily associated with pathways related to one-carbon metabolism and cellular stress responses, whereas ProBet shows a closer contextual association with signaling-related processes. By integrating experimental data, computational analyses, and human metabolomic information, this work supports the interpretation of betaines as biomarkers of dietary intake and systemic metabolic status.

## 1. Introduction

Betaines, which are derived from α-amino acids, constitute a heterogeneous class of quaternary ammonium compounds. These compounds are distinguished by the presence of a carbon-bound trimethylammonium center in an α position with respect to the carboxyl group of the amino acid. This structural arrangement confers a stable zwitterionic form, marked by a permanent positive charge on the nitrogen atom and a high degree of hydrophilicity. These features strongly influence their solubility, membrane permeability, and interactions with transporters and enzymatic systems [1,2].

Among betaines, glycine betaine (trimethyl glycine, GlyBet) is the most extensively studied, largely due to its central role in one-carbon metabolism. Acting as a methyl donor in the reaction catalyzed by betaine–homocysteine methyltransferase (BHMT), GlyBet contributes to methionine regeneration and supports the cellular pool of S-adenosylmethionine (SAM). Through this pathway, GlyBet participates in the regulation of plasma homocysteine—a well-established cardiovascular risk factor—and influences hepatic processes such as phosphatidylcholine synthesis and Very-Low-Density Lipoprotein (VLDL) secretion [3,4]. Beyond their role as methyl donors, many betaines also act as compatible osmolytes, helping to preserve protein structure and membrane integrity when cells face osmotic stress. This protective function is especially important in plants and microorganisms, and in animal organs that experience large osmotic shifts, most notably the kidney and the liver [5,6].

Over the past decade, our understanding of betaines has expanded: some derivatives show antioxidant or anti-inflammatory activity, while others—particularly aromatic and indolic species—are substrates for biotransformation by enzymes such as cytochrome P450 (CYP), monoamine oxidase (MAO), and catechol-O-methyltransferase (COMT). These metabolic routes raise the possibility that certain betaines could influence neuromodulatory pathways, a hypothesis that recent studies have begun to explore [7,8].

Notable examples include 4-butyrobetaine, the immediate precursor of carnitine via γ-butyrobetaine dioxygenase (BBOX1) and Organic Cation/Carnitine Transporter 2 (OCTN2) [9,10], and ergothioneine, a thiazole derivative with strong antioxidant activity and highly selective uptake mediated by Organic Cation/Carnitine Transporter 1 (OCTN1) [11].

Among dietary betaines, proline betaine (stachydrine, ProBet) deserves particular attention from a nutritional perspective. Unlike glycine betaine, which is both endogenously produced and diet-derived, ProBet is considered almost exclusively exogenous in humans and is highly enriched in citrus fruits and their derivatives. Its rapid absorption and efficient renal excretion make ProBet one of the most reliable objective biomarkers of citrus intake in nutrimetabolomic studies. Studying ProBet alongside GlyBet, therefore, offers a useful comparative framework to explore how structurally related betaines differ in dietary sources, metabolic handling, and biological interpretation, strengthening the nutritional relevance of the present work [12]. Structurally, ProBet’s cyclic scaffold imparts distinct physicochemical properties compared with glycine betaine, including greater conformational rigidity and a polarity profile that influences its absorption and interaction with specific transporters. Although ProBet functions primarily as an osmo-protectant in plants, emerging evidence points to potential biological activities—ranging from modest anti-inflammatory effects to possible influences on cardiovascular function—although these findings remain to be fully validated.

Diet is the primary source of exogenous betaines, and quantifying their levels in food matrices is therefore essential for understanding human exposure and its relationship with circulating metabolite pools.

Studies using HPLC-ESI-MS revealed that betaine levels in foods were highly variable, varying with species, plant tissue, and even growing conditions or agricultural practices [13,14]. This variability implies that dietary exposure to betaines can vary widely between individuals and populations, with possible implications for metabolic balance and nutritional assessment. A further layer of complexity emerges when experimental data are integrated with clinical metabolomics resources such as the Human Metabolome Database (HMDB). This publicly accessible repository aggregates thousands of human metabolic profiles generated on high-resolution analytical platforms and supplies quantitative data on metabolite levels in plasma, urine, and tissues, including various betaines [15]. Such data enable comparisons across physiological and pathological states and help identify patterns associated with age, nutritional status, renal function, and disease.

Despite the abundance of experimental and clinical data, several key questions remain unresolved. These include the structure–function relationships governing betaine activity, their bioavailability, the role of membrane transporters, and the identification of specific molecular targets. In this context, the adoption of computational strategies constitutes a pragmatic and complementary approach to experimentation. Computational approaches—such as structural curation, physicochemical descriptor calculation, Quantitative Structure–Activity Relationship (QSAR)-based model for ADMET (absorption–distribution–metabolism–excretion–toxicity) prediction, and similarity-driven target inference—offer a strategy for addressing these gaps [16,17,18,19,20]. The pkCSM platform encodes molecular structures as distance-based graph signatures and applies supervised machine-learning models trained on experimentally validated datasets to predict pharmacokinetic and toxicity-related properties [17,19,20].

These tools have steadily found their place in computational pharmacology and nutraceutical discovery, where they have proven valuable in early-stage screening of small molecules, natural products, and amino-acid derivatives, helping to prioritize compounds with favorable pharmacokinetic and safety profiles. This integrative strategy is particularly well suited to betaines, which share common structural motifs (a quaternary N^+^ center and a carboxylate side chain), have modest molecular weights, and therefore benefit from rapid triage for potential enzymatic or transporter interactions.

Similarity-based target-prediction tools, such as SuperPred (https://prediction.charite.de) (accessed on 1 December 2025), a platform developed at the Charité in Berlin for the Anatomical Therapeutic Chemical (ATC) classification of drugs and for the prediction of their molecular targets using machine learning models, have found practical use in nutraceutical research because they let researchers rapidly place a natural compound in the context of known pharmacology. These in silico suggestions do not replace in vitro or in vivo studies. Rather, they help narrow the field by generating focused, testable hypotheses about how bioactive natural products might interact with biological systems [21].

Structural-similarity approaches have proven their worth across many practical applications in the literature. For example, chemical-similarity-based classification has been reported to predict ATC categories with accuracies exceeding 80%, reinforcing the practical connection between molecular structure, likely targets, and clinical indication [21]. In this context, the “Indications of Predicted Targets” is used as an explanatory framework to contextualize similarity-based predictions within known biological and pharmacological spaces, rather than as evidence of direct drug-like activity. It is worth noting that several natural compounds—such as polyphenols, flavonoids, and various alkaloids—with structural similarity to established anti-inflammatory, antioxidant, or metabolic drugs have proven useful in generating testable hypotheses about molecular targets and pathways. However, for highly polar metabolites such as betaines, which lack classical pharmacophoric features and primarily exert indirect, metabolism-dependent effects, these predictions should be interpreted qualitatively.

Alterations in betaine metabolism have been repeatedly reported in a range of clinically relevant conditions, including nonalcoholic fatty liver disease (NAFLD) and its progression to nonalcoholic steatohepatitis (NASH), cardiovascular disease, and neuropsychiatric disorders. In these settings, changes in circulating glycine betaine levels have been associated with impaired one-carbon metabolism, oxidative stress, inflammation, and altered hepatic or renal function, highlighting their potential value as sensitive metabolic indicators rather than disease-specific markers. Despite this growing body of evidence, existing studies have largely focused on individual betaines in isolation, without a comparative framework that integrates dietary sources, systemic handling, and human metabolomic variability. In particular, glycine betaine and proline betaine differ markedly in their dietary origin, endogenous metabolism, and clearance, yet are often discussed separately or interchangeably in nutritional and clinical literature. The lack of an integrated evaluation hampers the interpretation of plasma and urinary betaine measurements and limits their use as objective nutritional biomarkers.

The present study addresses this gap by providing a side-by-side characterization of GlyBet and ProBet that combines targeted food analysis, in silico pharmacokinetic profiling, and integration with human metabolomic data. By jointly examining these two betaines, we aim to clarify their complementary roles as dietary exposure markers and to provide a coherent framework for interpreting betaine measurements in nutritional and metabolic research. Specifically, we combined experimental measurements, computational predictions, and metabolomic datasets to build a coherent, practical depiction of the two food-derived betaines—GlyBet and ProBet. First, we quantified GlyBet and ProBet across a variety of plant-based foods using HPLC-ESI-MS. We then examined their physicochemical properties and predicted ADMET profiles using in silico tools such as pkCSM to provide a comparative, qualitative description of their pharmacokinetic and safety-related features. We also used the SuperPred 3.0 web tool in an exploratory manner to contextualize these molecules with known and biological signaling spaces and to generate hypothesis-driven insights for mechanistic hypotheses, rather than to infer direct drug-like target interactions. Finally, by integrating these results with plasma concentration data from metabolomic repositories (including HMDB), we explored how dietary exposure relates to circulating levels of GlyBet and ProBet across different physiological and pathological conditions. Taken together, this layered approach was designed to clarify the nutritional and metabolic features of GlyBet and ProBet in order to support their potential use as biomarkers of dietary intake and metabolic status. This will help to decide which hypotheses are the most promising for testing in the future.

## 2. Materials and Methods

All reagents were of analytical grade or LCMS quality. Formic acid (99% purity) was provided by Merck (Sigma-Aldrich, Darmstadt, Germany). Ultrapure water (18.2 MΩ·cm resistivity) was produced using a Milli-Q system (Millipore, Burlington, MA, USA). Glycine Betaine (GlyBet) and Proline Betaine (ProBet) analytical standards were purchased from Cayman Chemical distributor (Vinci-Biochem Srl, Vinci, Firenze, Italy). Stock solutions were prepared at 2 mg·L^−1^ in 0.1% (*w*/*w*) formic acid and serially diluted to build calibration curves (0.001–0.2 mg·L^−1^). For each analytical batch, three quality-control levels (low, medium, high) were methodically prepared and analyzed.

### 2.1. Samples

A representative set of plant-derived food matrices was obtained from several local producers: selected vegetables (spinach, Sweet potato, Broccoli, Cauliflower), fruits (Peach, Pear, Strawberry, Cranberry, Kiwi) and citrus fruits (orange, mandarin, bergamot, bitter orange, chinotto) and flour of cereals and pseudocereals (quinoa, rye, barley, durum and soft wheat, millet, rice).

Fresh fruit samples were homogenized using an Ultra-Turrax™ Immersion Blender (Fisher Scientific, Segrate (MI), Italy). The samples were subjected to a processing speed of 10,000 rpm for a duration of 20 min. In order to ensure thermal stability and prevent potential degradation of the sample, the procedure was carried out at a controlled temperature using an ice bath. Subsequently, the homogenized samples were divided into aliquots and subsequently stored at −20 °C until analysis. For each matrix, three independent extractions (*n* = 3) were performed to evaluate reproducibility.

### 2.2. Extraction of Betaines from Food Matrices

Five hundred milligrams of homogenized material or flour was suspended in 2.5–5.0 mL of 0.1% formic acid (ratio 1:5 or 1:10 *w*/*w*, depending on the matrix). The suspension was placed on a magnetic stirrer and subjected to constant agitation at 500 rpm for a duration of 3 h at room temperature (RT). Subsequently, samples were centrifuged at 18,000× *g* for 60 min (Allegra X15R, Beckman Coulter, Brea, CA, USA), and the supernatants were filtered through 0.45 μm membranes (Whatman, Cytiva, Marlborough, MA, USA). This procedure was carried out using a benchtop centrifuge model, Allegra X15R, manufactured by Beckman Coulter (Brea, CA, USA). The clarified supernatants were sequentially filtered on 0.45 μm filters (Whatman, Cytiva, Marlborough, MA, USA). Extracts were stored in 500 μL aliquots at −20 °C. Matrix effects were evaluated by standard addition (spike-recovery), with acceptable recoveries set between 80% and 120%.

### 2.3. Analysis and Quantification by HPLC-ESI-MS

The Quantification of betaines was performed on an LCMSD SL (Liquid Chromatography Mass Spectrometry Detector Single Quadrupole) Agilent 1100 HPLC system (Agilent Technologies, Santa Clara, CA, USA) operating in positive ESI mode according to Servillo et al. [13,14]. The separation process was carried out on a Supelco Discovery C8 column (250 × 3.0 mm, 5 μm) under isocratic conditions, employing 0.1% formic acid as mobile phase. The flow rate was set at 100 μL·min^−1^, and the injection volume was 10 μL. The identification of GlyBet and ProBet was based on two factors: their retention time and the monitoring of selected characteristic ions. Specifically, ions at *m/z* 118.1 and *m/z* 59 were monitored for GlyBet and ions at *m/z* 114 and *m/z* 84 for ProBet, with parameters optimized by direct infusion of analytical standards. The quantification was obtained by comparison with external calibration curves; linearity was tested on six levels with R^2^ > 0.99. The Limit of Detection (LOD) and Limit of Quantification (LOQ) were determined experimentally (S/N = 3 and S/N = 10, respectively). Results are reported as mg·kg^−1^ fresh weight for fruits and vegetables and as mg·kg^−1^ as-is for cereal and pseudocereal flours, and are expressed as mean ± SD of triplicates. For both GlyBet and ProBet, the LOQ was below 0.1 mg/kg for all matrices analyzed.

### 2.4. Structural Curation and Calculation of Descriptors

Chemical structures of GlyBet and Probet were represented as canonical SMILES and curated by removing salts/solvents, assigning the predominant protonation state at pH 7.4 and normalizing tautomers. We calculated standard physicochemical descriptors for each compound: molecular weight (MolWt), topological polar surface (TPSA), estimated partition coefficient (cLogP), number of hydrogen bond donors and acceptors (HBD, HBA), number of rotatable bonds (RotB), number of heavy atoms, and aromatic ring count. All descriptor calculations were performed using the open-source cheminformatics toolkit RDKit (http://www.rdkit.org) (accessed on 1 December 2025).

### 2.5. ADMET and Molecular Target Predictions

ADMET profiles were predicted from curated SMILES using pkCSM (https://biosig.lab.uq.edu.au/pkcsm/) (accessed on 1 December 2025) [19,20]. Predicted endpoints included aqueous solubility, Caco-2 permeability, estimated intestinal absorption, skin permeation (log Kp), P-gp interactions, volume of distribution (VDss), plasma free fraction, Blood–brain barrier (BBB) and Central Nervous System (CNS) permeation (logBB, logPS), Cytochromes (CYPs) substrate/inhibitor status (CYP1A2, CYP2C9, CYP2C19, CYP2D6, CYP3A4), total clearance, Organic Cation Transporter 2 (OCT2) substrate status, AMES mutagenicity, oral LD50, Lowest-Observed-Adverse-Effect Level (LOAEL), hepatotoxicity and skin sensitization. Numerical outputs were categorized to qualitative classes (favorable/indeterminate/critical) using predefined thresholds (for example, logBB < −0.1 was considered low BBB penetration). Potential molecular targets were predicted with SuperPred web tool according to Nickel et al. [18,21], accessible via the web at the following link https://prediction.charite.de (accessed on 1 December 2025), and similarity scores and functional classes were recorded (enzyme, transporter, receptor). All in silico results were treated as hypothesis-generating and interpreted in the context of existing literature.

### 2.6. Metabolomic Databases and Correlation with Human Plasma Levels

Physiological and Clinical metabolomic data for GlyBet and ProBet were retrieved from Human Metabolome Database (HMDB; https://hmdb.ca) (accessed on 1 December 2025) [15] and selected publications; only datasets reporting complete quantitative statistics (mean, SD, range) and a clear clinical definition were included. We considered conditions such as healthy controls, renal impairment, metabolic disorders, cardiovascular disease, neuropsychiatric disorders and pregnancy. Plasma concentrations were harmonized to common units (μmol·L^−1^ or mg·L^−1^) and compared with dietary intake estimates derived from food quantification to explore correlations between intake and circulating pools.

### 2.7. Multiple Regression Model for Plasma GlyBet

All predictor variables were coded according to their nature before model fitting, with continuous variables entered as such and categorical clinical conditions encoded as binary indicators. Because the regression model was constructed using aggregated mean values derived from published studies and metabolomic databases, formal model validation procedures such as cross-validation, residual diagnostics, or bootstrapping were not applicable in a strict statistical sense. Differences in study design, population characteristics, analytical platforms, and sample sizes introduce unavoidable heterogeneity that limits the interpretability of coefficient estimates. Accordingly, the model should be regarded as descriptive and exploratory, providing qualitative insights into patterns of association rather than robust or unbiased effect size estimates. We started exploring the factors that determine plasma GlyBet levels (μmol·L^−1^) in physiological and pathological states, collecting mean concentration values from the HMDB and published studies. The selection of predictors was based on a comprehensive review of the existing literature. The following factors were considered: dietary GlyBet intake, kidney function, pregnancy status, the presence of rare metabolic diseases or metabolic syndromes (e.g., NAFLD: non-alcoholic fatty liver disease) or NASH [non-alcoholic steatohepatitis]), and cardiovascular disease [22,23,24,25,26,27,28,29]. Coefficient significance was tested with *t*-test, and overall model fit was assessed by analysis of variance (ANOVA, with a confidence level set at 95%), acknowledging that statistical inference is limited to study-level-association.

## 3. Results and Discussion

### 3.1. Analytical Performance and Method Validation

We quantified GlyBet and ProBet by HPLC ESI-MS using a single-quadrupole mass spectrometer, confirming compound identity by matching retention times and characteristic in-source fragmentation patterns to certified standards. Calibration curves were linear for both analytes (r^2^ > 0.99). Intraday precision was acceptable (CV% < 15% for medium and high QC levels) and spike recovery experiments returned values between 80% and 120%, supporting the method’s suitability as a quantitative method for the assessment of these compounds in food matrices.

### 3.2. Dietary Distribution of GlyBet and ProBet

The quantified content of GlyBet and ProBet and their range of variability in different food matrices are shown in Table 1. Based on our results, the two betaines showed clearly different dietary patterns. GlyBet was abundant in leafy vegetables and present at appreciable levels in flour of several cereals and pseudocereals. Raw spinach, in fact, exhibited the highest concentrations, on the order of grams per kilogram, consistent with its known capacity to accumulate this osmolyte. In cereals and pseudocereals flours, we measured intermediate GlyBet concentrations, while common fruits typically contained only trace amounts (approximately 1–3 mg·kg^−1^) (Table 1).

ProBet followed a distinct distribution, concentrating mainly on Citrus Genus fruits. Measured values in bergamot, chinotto and bitter orange ranged from about 500 to 650 mg·kg^−1^, in line with previous reports that identify ProBet as a characteristic marker of these matrices and their derivatives [13,30]. However, in most cereal and pseudocereal flours, ProBet content showed relatively low levels, ranging from 0.2 mg·kg^−1^ to 4 mg·kg^−1^ in flours of buckwheat and barley (Table 1). The variability observed across samples agrees with earlier surveys showing wide variation in betaine content across plant species and highlights important sources of heterogeneity: botanical species, agronomic practices, ripeness at harvest and post-harvest handling all influenced betaine content [13,14]. These factors should be considered when estimating population-level intakes. Together, these observations reinforce the idea that dietary exposure to betaines can differ markedly between individuals and populations. A limitation of this study is that food sampling did not capture the full variability in betaine content related to species, growing conditions, or post-harvest handling, since each food item was obtained from a single commercial source, and therefore, the reported values should be interpreted as indicative rather than as comprehensive estimates of dietary exposure. Nevertheless, our results provide a quantitative foundation for estimating dietary exposure from common plant foods and for designing follow-up studies aimed at linking intake with circulating levels and biologically relevant metabolic outcomes.

### 3.3. ADMET Computational Prediction

We performed in silico ADMET analyses for GlyBet and ProBet to obtain an integrated, predicted pharmacokinetic and safety profile. The results of the in silico ADMET predictions for GlyBet and ProBet are illustrated in Table 2. Overall, GlyBet and ProBet show largely overlapping predicted ADMET profiles, consistent with their identity as small, hydrophilic osmolytes, but they differ meaningfully in aqueous solubility and in predicted risk of skin sensitization.

Both compounds are predicted to have high oral absorption (predicted human intestinal absorption = 100%), and their Caco2 permeability estimates are very similar (log Papp ≈ 1.28 × 10^−6^ cm/s). It should be noted that Caco-2-based permeability estimates and in silico absorption predictions do not account for the presence of the intestinal mucus layer. This limitation is particularly relevant for highly polar and zwitterionic compounds such as betaines and should be considered when interpreting predicted absorption values, which are intended to provide qualitative and comparative insights rather than quantitative estimates of bioaccessibility. GlyBet is predicted to be more water-soluble (log mol L^−1^ 0.434) than ProBet (log mol L^−1^ −0.097), which could translate into faster dissolution and potentially quicker onset after oral dosing. Skin permeability estimates are low for both (log Kp ≈ −2.89), indicating limited passive dermal absorption. Distribution predictions indicate modest tissue distribution for both molecules, with ProBet showing a slightly higher steady-state volume of distribution (VDss log L/kg 0.188 versus 0.008 for GlyBet) and both compounds having high predicted fractions unbound in plasma (Fu ≈ 0.74–0.77). Blood–brain barrier penetration and CNS permeability are predicted to be low (log BB < 0; log PS ≈ −2.4 to −2.6), suggesting a low likelihood of central nervous system effects and supporting a predominantly peripheral mode of action [19,20,31]. In the context of food science, skin sensitization predictions should be interpreted solely as general safety descriptors derived from in silico ADMET profiling. These predictions do not reflect dermal exposure following oral intake of betaine-containing foods, which was not assessed in this study and is expected to be negligible due to the high polarity, limited tissue distribution, and rapid renal clearance of both betaines. Accordingly, this information is included to support a qualitative comparison of safety profiles rather than to imply dietary risk. While betaines are generally considered mild compounds, some may act as skin sensitizers. In this regard, GlyBet was predicted to be potentially skin sensitizing, whereas ProBet was not, suggesting a more favorable dermal tolerability profile for ProBet. This distinction may be relevant for the selection of specific betaines in consumer products, particularly for individuals with sensitive skin.

Metabolism predictions are favorable: neither compound is predicted to be a substrate or inhibitor of the major cytochrome P450 enzymes tested (CYP1A2, 2C9, 2C19, 2D6, 3A4), which implies a low risk of CYP-mediated drug–drug interactions. The data are consistent with the limited affinity that highly polar molecules show for lipophilic catalytic sites typical of CYPs [17,19,20]. That said, these in silico results do not exclude the involvement of phase II conjugation pathways or non-CYP enzymes in biotransformation, so experimental metabolic profiling remains advisable. Excretion predictions point to renal clearance as an important elimination route; ProBet has a higher predicted total clearance (log mL min^−1^ kg^−1^ 0.638) than GlyBet (0.352), indicating faster elimination of the proline derivative. Predictions also suggest no potential interactions with renal SLC transporters, while P-glycoprotein is not expected to play a major role in intestinal efflux or in limiting CNS access [19,20,21,32,33].

Consequently, renal function and transporter expression could influence systemic exposure to these betaines.

Safety predictions are reassuring overall: both compounds are predicted to be negative in AMES mutagenicity and not hepatotoxic. They are also predicted to not inhibit hERG I or II channels, although methodological uncertainties and limitations of representativeness of datasets remain and require experimental confirmation by standard tests [19,20].

### 3.4. Physiological Variability of GlyBet and Alterations in Pathological Conditions

**Figure 1:**
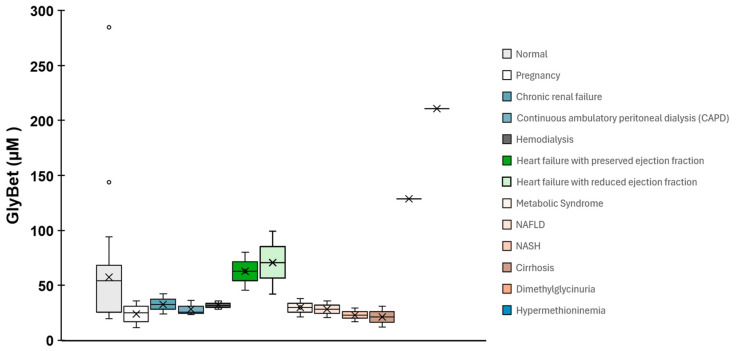
Boxplot comparing plasma GlyBet (µM) distributions across physiological and pathological conditions. Each box shows median, interquartile range, whiskers and outliers. Metabolic disorders (hypermethioninemia, dimethylglycinuria) display the highest GlyBet values; heart failure and metabolic syndrome show moderate increases; and pregnancy, NAFLD/NASH and cirrhosis are associated with lower levels. Renal conditions (chronic renal failure, CAPD, hemodialysis) exhibit wide variability, reflecting the strong influence of kidney function on betaine clearance. Overall, the pattern highlights GlyBet’s sensitivity to metabolic and renal disturbances and its potential utility as a biomarker. Data were compiled from published clinical and metabolomic studies and from the Human Metabolome Database (HMDB); glycine betaine is listed in HMDB under HMDB0000043. Primary literature sources used to assemble the dataset and annotate group labels: Abdelmalek et al., 2009 [38], Bahado Singh et al., 2012; 2014 [22,23]; Barić et al., 2004 [24]; Sookoian et al., 2017 [39]; Corbin et al., 2012 [37]; Kalhan et al., 2011 [35]; Koike et al., 2014 [25]; Laryea et al., 1998 [34]; Lever et al., 1994; 2005 [36,40]; McGregor et al., 2001 [26]; Psychogios et al., 2011 [27]; Moolenaar et al., 1999 [28]; Zordoky et al., 2015 [29].

GlyBet is a key methyl-group donor in the methionine cycle and an important osmoprotective molecule. Its circulating concentrations display wide physiological variability and are influenced by age, dietary intake, renal function, and overall metabolic status [22,23,24,25,26,27,28,29]. This variability becomes more pronounced in pathological conditions, where plasma GlyBet levels shift in characteristic patterns. Altered concentrations have been reported in rare metabolic disorders [24,28], kidney disease [26], cardiovascular dysfunction [29], neuropsychiatric conditions such as schizophrenia [25], and complex physiological states including pregnancy and fetal development [22,23] (Figure 1). Collectively, these observations support the interpretation of GlyBet as a sensitive biomarker of metabolic balance.

In healthy individuals, plasma GlyBet concentrations span a broad range, typically between 20 and 285 μM, with mean values generally between 33 and 72 μM. Cohort studies report average concentrations of 31.4 μM [27] and 33.6 μM [26], while Laryea et al. [34] described a reference interval of 20–144 μM. The consistently large interindividual variability observed across healthy cohorts reflects the dynamic regulation of methylation processes and cellular osmotic balance, as well as genetic, environmental, and methodological influences highlighted in metabolomic studies [25,27] (Figure 1).

Pregnancy represents a distinct physiological state characterized by systematically lower GlyBet concentrations compared with non-pregnant healthy populations, likely reflecting increased maternal–fetal utilization and altered methyl-donor metabolism [22,23]. During the first trimester, mean plasma concentrations typically fall in the range of approximately 18–22 μM, with modest but reproducible deviations reported in pregnancies complicated by fetal chromosomal abnormalities or preeclampsia [22,23]. These findings underscore the sensitivity of GlyBet to physiological metabolic redistribution during early gestation.

When comparing physiological and pathological states (Figure 1), coherent trends emerge across conditions. It should be noted that for rare metabolic disorders, available plasma GlyBet data are often derived from single-patient reports or very small cohorts. While these conditions provide valuable insight into extreme perturbations of one-carbon metabolism, limited sample sizes constrain the robustness of quantitative comparisons and preclude definitive mechanistic inference. Accordingly, observations in these groups are presented in a descriptive and illustrative manner, with an emphasis on phenomenological patterns rather than causal interpretation. Similar considerations apply, to varying degrees, to other conditions represented by heterogeneous metabolomic datasets with limited original sample sizes. Rare metabolic disorders such as hypermethioninemia and dimethylglycinuria display the highest reported GlyBet concentrations, consistent with endogenous accumulation resulting from enzymatic defects in one-carbon metabolism [24,28]. Renal impairment is generally associated with reduced or low–normal GlyBet values, reflecting altered clearance and metabolic disturbances typical of uremic conditions [26]. Cardiovascular diseases and schizophrenia tend to show GlyBet concentrations toward the upper range of normal, consistent with broader metabolic alterations reported in these conditions [25,29].

In metabolic liver disease, plasma GlyBet concentrations are frequently reduced. Multiple metabolomic studies report lower levels in NAFLD and a further decline in NASH, with values consistently lower than those observed in healthy controls [35]. Similar reductions have been described in metabolic syndrome [36] and liver cirrhosis [37], where large standard deviations reflect substantial metabolic heterogeneity in advanced disease. These consistent observational patterns support the interpretation of GlyBet depletion as a marker of impaired methylation capacity and altered hepatic metabolism in progressive liver disease.

Therapeutic betaine supplementation has been explored as a strategy to restore methyl-donor availability and improve hepatic metabolic balance. Clinical studies report normalization of plasma GlyBet levels and improvement in hepatic steatosis following supplementation, although effects on fibrosis remain inconsistent and dependent on study design and disease stage [38,39]. Overall, the available evidence supports the view of GlyBet primarily as a biomarker of metabolic stress, while its potential modulatory role in disease progression remains context-dependent and requires targeted experimental validation.

### 3.5. Correlation Between Plasma GlyBet Levels with Dietary Intake and Plasma Levels

Dietary intake estimates of GlyBet were derived from published nutritional and metabolomic studies reporting habitual consumption patterns, dietary records, or biomarker-based intake assessments, rather than being calculated directly from the food concentration data generated in the present study. Reported intake values were harmonized to common units (mmol/day) and used at an aggregated study-level to explore broad associations with circulating plasma GlyBet concentrations. This approach was adopted for exploratory purposes and is intended to provide qualitative insights into relationships between dietary exposure and systemic betaine levels, rather than precise estimates of individual intake. Food concentration data reported in Table 1 are provided to characterize dietary sources of betaines and were not used to calculate the dietary intake estimates applied in the regression analysis.

Understanding how plasma GlyBet concentrations relate to dietary intake is essential for contextualizing its role as a nutritional and metabolic biomarker. The wide interindividual variability observed in plasma GlyBet levels reflects the combined influence of dietary intake, intestinal absorption, hepatic metabolism, and renal clearance. Although intestinal absorption of GlyBet is generally efficient, only a fraction of the ingested amount contributes to circulating pools, as a substantial portion is utilized in hepatic methylation reactions. Nevertheless, dietary intake remains an important determinant of physiological variability, particularly in individuals with high consumption of GlyBet-rich foods. To further examine the interaction between dietary exposure and pathophysiological status, a multiple regression model was developed integrating dietary GlyBet intake and major clinical conditions known to influence plasma GlyBet levels. Because the model was built using aggregated mean concentrations derived from heterogeneous studies and metabolomic platforms, the estimated coefficients should be interpreted strictly as study-level associations rather than individual-level effect sizes. Accordingly, the analysis is exploratory and hypothesis-generating.

The regression model is summarized as follows:

$${Serum\;GlyBet}_{\left(uM\right)}=\beta_{0}+\beta_{1}Dietary\;\mathrm{intake}+\sum_{i=k}^{n}\beta_{k}\;{Condition}_{k}+\epsilon$$

**Figure 2:**
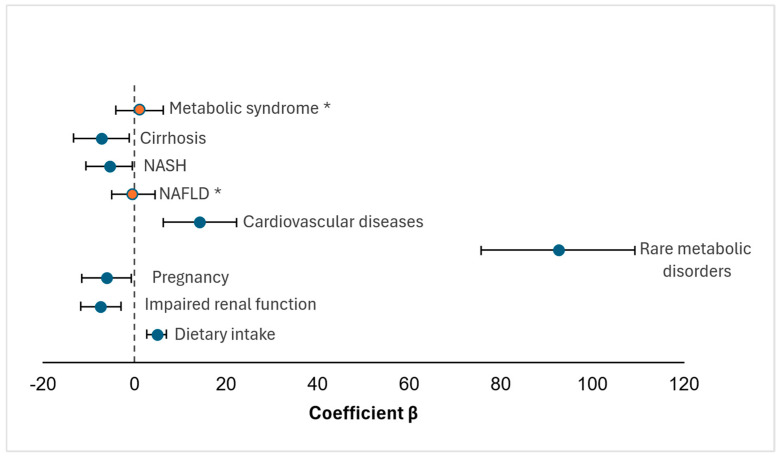
Forest plot of regression coefficients (β) from an exploratory study-level association model for predictors of plasma glycine betaine (GlyBet), with 95% confidence intervals. Horizontal bars represent the 95% confidence intervals for each coefficient. Predictors that were not statistically significant (*p* ≥ 0.05) are shown in orange and marked with an asterisk (*). Specifically, nonalcoholic fatty liver disease (NAFLD; β = −0.3, 95% CI [−5.00, 4.40], *p* = 0.890) and metabolic syndrome (β = +1.1, 95% CI [−4.18, 6.38], *p* = 0.680) did not show significant associations with plasma GlyBet concentrations. The vertical reference line at β = 0 indicates the null effect.

Despite these limitations, the model demonstrated overall statistical significance (*p* < 0.001) and provided a coherent description of the compiled data. The results, summarized in Table 3 and Figure 2, highlight the multifactorial nature of plasma GlyBet regulation, reflecting the interplay between nutritional intake and clinical conditions.

Dietary GlyBet intake showed a positive association with aggregated plasma concentration estimates (β_1_ = +4.85, SE = 1.10), indicating that, at the group level, an increase of 1 mmol/day in intake corresponds to an approximate 5 μM increase in circulating GlyBet. This observation is consistent with prior nutritional and metabolomic evidence [27]. Among clinical variables, impaired renal function (β_2_ = −7.40, SE = 2.25, *p* = 0.002) and pregnancy (β_3_ = −6.10, SE = 2.80, *p* = 0.030) were significantly associated with lower aggregated plasma GlyBet estimates, consistent with altered clearance and increased methyl-donor utilization, respectively [22,23,26].

Rare metabolic disorders exhibited the largest positive association with plasma GlyBet (β_4_ = +92.5, SE = 8.6), reflecting endogenous accumulation linked to enzymatic defects in one-carbon metabolism [24,28]. Cardiovascular diseases were also associated with higher aggregated GlyBet estimates (β_5_ = +14.2, SE = 4.1, *p* = 0.001), consistent with metabolic alterations reported in these conditions [29]. In contrast, NAFLD and metabolic syndrome were not significantly associated with plasma GlyBet at the group level, whereas NASH and cirrhosis showed significant negative associations, consistent with advanced hepatic dysfunction and impaired methylation capacity.

Overall, these findings indicate that aggregated plasma GlyBet concentration estimates reflect a complex biomarker influenced by both dietary exposure and pathophysiological factors. While dietary intake contributes meaningfully to circulating levels, a substantial proportion of variability is driven by clinical conditions that affect GlyBet metabolism and clearance. Consequently, plasma GlyBet measurements require contextualized interpretation that accounts for both nutritional and metabolic determinants.

### 3.6. GlyBet as a Context-Dependent Metabolic Modulator

Similarity-based target prediction analyses were included exclusively to support exploratory, hypothesis-generating interpretation. Given the highly polar nature of betaines and the absence of classical pharmacophoric features, predicted associations should not be interpreted as evidence of direct molecular interactions, but rather as contextual links to biological pathways reported in the literature.

Beyond its established role as a methyl-group donor in the methionine cycle, GlyBet has been described as a sensitive metabolic node that responds to oxidative stress, alterations in one-carbon metabolism, hepatic dysfunction, and changes in renal handling—factors that collectively influence its systemic distribution. Clinical and metabolomic studies consistently associate reduced plasma GlyBet levels with impaired homocysteine remethylation, increased inflammatory burden, and the progression of metabolic disorders, such as NAFLD, NASH, and cardiovascular disease [40,41,42]. These observations suggest that GlyBet primarily reflects underlying metabolic stress, while its potential contribution to pathophysiological processes remains context-dependent.

Within this framework, predicted and literature-supported pathway associations converge on processes involved in inflammation and cellular stress responses (Table 4). Among these, the NF-κB signaling axis represents a well-characterized example. Experimental studies in cellular and animal models indicate that GlyBet supplementation is associated with attenuation of NF-κB activation and reduced expression of pro-inflammatory mediators, effects that have been attributed to indirect modulation of redox balance, endoplasmic reticulum stress, and methyl-donor availability rather than to direct ligand–target interactions [36,37,43,44]. These findings provide a plausible biological context for the inverse association between plasma GlyBet levels and inflammatory markers observed in metabolic liver disease.

A second illustrative pathway concerns lysosomal homeostasis, particularly in relation to cathepsin D (CTSD). GlyBet functions as an osmoprotectant capable of stabilizing proteins and membranes, a property that becomes relevant under metabolic stress conditions such as NAFLD/NASH. Preclinical studies suggest that GlyBet depletion is associated with lysosomal membrane permeabilization and altered CTSD localization, whereas supplementation restores lysosomal integrity, supports autophagic flux, and reduces aberrant extracellular release of CTSD [45,46,47]. Although these observations are derived primarily from experimental models, they are consistent with clinical reports linking reduced GlyBet levels to markers of lysosomal dysfunction in advanced liver disease.

From a translational perspective, these data support the interpretation of GlyBet primarily as a biomarker of metabolic and inflammatory stress, while its potential modulatory role in pathways such as NF-κB signaling and lysosomal regulation remains hypothesis-generating. Although betaine supplementation has shown beneficial effects on hepatic steatosis in some clinical studies, evidence for broader disease-modifying effects remains heterogeneous and dependent on dosage, disease stage, and study design [38,40]. Accordingly, mechanistic interpretations should be viewed as contextual frameworks that warrant targeted experimental and clinical validation rather than definitive causal models.

Based on the available human and experimental evidence, alterations in plasma glycine betaine levels are best interpreted as downstream markers of metabolic and hepatic dysfunction rather than as causal drivers of disease pathogenesis. Changes in GlyBet concentrations primarily reflect variations in methyl-group demand, oxidative stress burden, hepatic metabolic capacity, and renal handling. While experimental studies indicate that betaine availability may modulate inflammatory and stress-related pathways under specific conditions, these effects appear to be indirect, context-dependent, and insufficient to support a primary causal role in human disease. Accordingly, in clinical and nutritional settings, GlyBet should be regarded as an integrative metabolic biomarker rather than a disease-modifying agent.

### 3.7. ProBet: Dietary Biomarker and Context-Dependent Signaling Associations

Unlike GlyBet, ProBet is considered almost exclusively exogenous in humans [40]. This characteristic has established ProBet as one of the most robust and specific objective biomarkers of citrus intake in nutrimetabolomic studies, consistently outperforming self-reported dietary assessments [30]. Following citrus consumption, ProBet is rapidly absorbed, reaches peak plasma concentrations within 1–2 h, and is efficiently eliminated by the kidney, with 80–90% of the ingested dose excreted unchanged in urine within 24 h [30,40,48]. Because it is neither synthesized endogenously nor retained in tissues, circulating and urinary ProBet levels reflect recent dietary exposure, providing a precise temporal window for intake assessment [48,49].

Beyond its established role as a dietary biomarker, limited experimental evidence suggests that ProBet may exert context-dependent biological effects. Similarity-based target prediction analyses were therefore used in an exploratory, hypothesis-generating manner, with the explicit caveat that predicted associations do not imply direct ligand–target interactions. Given the highly polar nature of ProBet, such predictions are best interpreted as contextual links to biological pathways rather than mechanistic evidence (Table 5).

Within this framework, the ERK/MAPK signaling axis emerged as a recurring point of convergence between in silico predictions and experimental observations. In endothelial cell models, ProBet has been reported to modulate ERK signaling in a context-dependent manner, with effects ranging from activation of VEGFR2–ERK pathways under physiological conditions to attenuation of ERK phosphorylation in models of oxidative stress and inflammation [50]. These findings are consistent with a redox-mediated modulation of MAPK signaling rather than a direct receptor-mediated mechanism.

Other predicted pathway associations—including inflammatory signaling and transcriptional regulation—should be regarded as speculative and interpreted cautiously. While ProBet possesses antioxidant properties and may indirectly influence inflammatory pathways, current evidence remains limited and largely derived from experimental models. Importantly, citrus intake has also been shown to increase circulating glycine betaine levels [51], suggesting that some reported biological effects attributed to ProBet may reflect indirect interactions within broader methyl-donor and redox networks rather than ProBet-specific mechanisms.

Overall, the available evidence supports the interpretation of ProBet primarily as a highly specific biomarker of citrus intake, with emerging but still preliminary indications of context-dependent signaling associations. These observations warrant further investigation in well-controlled experimental systems but do not detract from ProBet’s primary value as a nutritional exposure marker in human studies.

## 4. Conclusions

In conclusion, this integrated study provides a coherent, evidence-based characterization of two dietary betaines, GlyBet and ProBet, by combining targeted LC–MS quantification, in silico ADMET profiling, and similarity-driven target prediction. Empirical data highlight markedly different dietary distributions—GlyBet being concentrated in leafy vegetables and cereals, and ProBet enriched in citrus fruits—while computational analyses indicate broadly overlapping peripheral pharmacokinetic profiles alongside divergent biological associations. Overall, the present findings support GlyBet and ProBet primarily as sensitive nutritional and metabolic biomarkers that integrate dietary exposure with hepatic and renal function. While experimental studies suggest that betaine availability may influence cellular stress and inflammatory pathways under specific conditions, the evidence presented here does not support a primary causal or disease-modifying role in humans. Accordingly, interpretations of betaine-related biological effects should remain contextual and hypothesis-generating, and future studies should focus on clarifying their utility as biomarkers rather than as direct therapeutic agents.

## Figures and Tables

**Table 1:** Quantification of Glycine Betaine (GlyBet) and Proline Betaine (ProBet) in Selected Foods by LC–MS. Concentrations are expressed as mg/kg fresh weight for fruits and vegetables, and as mg/kg as-is for cereal and pseudocereal flours.

	GlyBet (mg·kg^−1^ ± SD)	ProBet (mg·kg^−1^ ± SD)
CEREALS AND PSEUDOCEREAL FLOURS		
Buckwheat	10 ± 5	4 ± 2
Quinoa	611 ± 15	1 ± 1
Barley	255.0 ± 12	2 ± 1
Durum wheat	245.0 ± 16	0.6 ± 0.5
Soft wheat	180.0 ± 18	0.2 ± 0.2
Rye	310.0 ± 21	3 ± 1
Millet	132.0 ± 18	0.2 ± 0.1
Rice	8.4 ± 5	3 ± 0.7
FRUITS		
Bergamot	2.4 ± 0.4	600 ± 50
Chinotto	2.2 ± 0.3	500 ± 40
Cranberry	2.1 ± 0.5	50 ± 10
Grapefruit	2.0 ± 0.4	100 ± 15
Kiwi	1.5 ± 0.5	30 ± 15
Lemon	2.4 ± 0.4	80 ± 10
Mandarin	3.0 ± 0.4	150 ± 20
Orange	2.2 ± 0.5	200 ± 25
Bitter orange	3.3 ± 0.9	650 ± 50
Peach	1.0 ± 0.2	10 ± 3
Pear	12.0 ± 1.2	8 ± 6
Strawberry	1.8 ± 0.3	11 ± 6
VEGETABLES		
Raw spinach	5998 ± 151	22 ± 14
Sweet potato	307.6 ± 41	34 ± 10
Broccoli	1.1 ± 0.6	0.1 ± 0.1
Cauliflower	1.1 ± 0.8	0.1 ± 0.1

**Table 2:** In silico evaluation of the ADMET properties (Absorption, Distribution, Metabolism, Excretion, and Toxicity) of GlyBet and ProBet. The data was generated using the pkCSM platform, based on machine learning algorithms and graphical molecular signatures for predicting the pharmacokinetic and toxicological profile.

Model Name	Unit	GlyBet	ProBet
ADSORPTION			
Water solubility	Numeric (log mol L^−1)^	0.434	−0.097
Caco2 permeability	Numeric (log Papp in 10^−6^ cm/s)	1.285	1.282
Intestinal absorption (human)	Numeric (% Absorbed)	100	100
Skin Permeability	Numeric (log Kp)	−2.891	−2.899
P-glycoprotein substrate	Categorical (Yes/No)	Yes	Yes
P-glycoprotein I inhibitor	Categorical (Yes/No)	No	No
P-glycoprotein II inhibitor	Categorical (Yes/No)	No	No
DISTRIBUTION			
VDss (human)	Numeric (log L/kg)	0.008	0.188
Fraction unbound (human)	Numeric (Fu)	0.772	0.743
BBB permeability	Numeric (log BB)	−0.148	−0.131
CNS permeability	Numeric (log PS)	−2.449	−2.605
METABOLISM			
CYP2D6 substrate	Categorical (Yes/No)	No	No
CYP3A4 substrate	Categorical (Yes/No)	No	No
CYP1A2 inhibitor	Categorical (Yes/No)	No	No
CYP2C19 inhibitor	Categorical (Yes/No)	No	No
CYP2C9 inhibitor	Categorical (Yes/No)	No	No
CYP2D6 inhibitor	Categorical (Yes/No)	No	No
CYP3A4 inhibitor	Categorical (Yes/No)	No	No
EXCRETION			
Total Clearance	Numeric (log mL min^−1^ kg^−1^)	0.352	0.638
Renal OCT2 substrate	Categorical (Yes/No)	No	No
TOXICITY			
AMES toxicity	Categorical (Yes/No)	No	No
Max. tolerated dose (human)	Numeric (log mg kg^−1^ day^−1^)	1.048	0.811
hERG I inhibitor	Categorical (Yes/No)	No	No
hERG II inhibitor	Categorical (Yes/No)	No	No
Oral Rat Acute Toxicity (LD50)	Numeric (mol/kg)	2.006	2.142
Oral Rat Chronic Toxicity (LOAEL)	Numeric (log mg kg^−1^_bw day^−1^)	0.098	0.202
Hepatotoxicity	Categorical (Yes/No)	No	No
Skin Sensitisation	Categorical (Yes/No)	Yes	No
*T. Pyriformis* toxicity	Numeric (log ug L^−1^)	−0.442	−0.333
Minnow toxicity	Numeric (log mM)	2.895	2.804

**Table 3:** Estimated Regression Coefficients, Standard Errors, Confidence Intervals, and *p* Values for Predictors of Plasma Glycine Betaine from an exploratory study-level association model.

Independent Variable	Symbol	Coefficient β	Standard Error	*p*-Value
Intercept (baseline GLYBET)	β_0_	28.7	3.2	
Dietary intake of GLYBET (mmol/day)	β_1_	+4.85	1.10	
Impaired renal function	β_2_	−7.40	2.25	0.002
Pregnancy	β_3_	−6.10	2.80	0.030
Rare metabolic disorders	β_4_	+92.5	8.6	
Cardiovascular diseases	β_5_	+14.2	4.1	0.001
NAFLD	β_6_	−0.3	2.4	0.890
NASH	β_7_	−5.5	2.6	0.038
Cirrhosis	β_8_	−7.2	3.1	0.021
Metabolic Syndrome	β_9_	+1.1	2.7	0.680

**Table 4:** Predicted molecular targets of betaine identified through the SuperPred platform.

Molecular Target	Functional Class	Probability (%)	Model Accuracy (%)
NF-κB p105 subunit	Receptor	93	96
Bloom syndrome protein	Enzyme	89	96
CLK4	Enzyme	89	96
MAPK ERK2	Enzyme	88	96
Cathepsin D	Enzyme	79	94

**Table 5:** Predicted Molecular Targets of ProBet (SuperPred Platform).

Target Name	Functional Class	Probability	Model Accuracy
MAP kinase ERK2	Enzyme	100%	84%
Bloom syndrome protein	Enzyme	97%	70%
NF-κB p105 subunit	Receptor	90%	96%
Cannabinoid CB2 receptor	Receptor	87%	97%
Transcription intermediary factor 1-alpha	Transcriptional Coactivator/Coregulator	84%	96%
Cytochrome P450 3A4	Enzyme	83%	91%
Phosphodiesterase 3A	Enzyme	82%	93%
Casein kinase II alpha/beta	Enzyme	80%	99%

## Data Availability

The original contributions presented in this study are included in the article. Further inquiries can be directed at the corresponding author.
